# Translation and psychometric evaluation of the Spanish version of the problem areas in diabetes–pediatric version (PAID-Peds) survey

**DOI:** 10.1186/s13098-023-01199-3

**Published:** 2023-10-30

**Authors:** Josep-Oriol Casanovas-Marsal, Elisa Civitani Monzón, M. Pilar Ferrer Duce, Marta Ferrer Lozano, Marta Vara Callau, Delia González de la Cuesta, Rosa Yelmo Valverde, Victoria Pérez Repiso, Irune Goicoechea Manterola, Antonio de Arriba Muñoz

**Affiliations:** 1 Instituto Investigación Sanitaria Aragón, Avda. San Juan Bosco 13, Zaragoza, 50009, Spain; 2 Hospital Universitario Miguel Servet, Avenida Isabel la Católica 1-3, Zaragoza, 50009, Spain; 3 Hospital Universitario Ramon y Cajal, Carretera Colmenar Viejo km. 9100 Madrid, 28034, Spain; 4 Hospital Sant Joan de Déu, Passeig Sant Joan de Déu 2, Barcelona, 08950 Spain

**Keywords:** Type 1 diabetes mellitus, Quality of life, Reproducibility of results, Health surveys

## Abstract

**Background:**

Metabolic control and psychological management of paediatric type 1 diabetes mellitus (T1DM) can be challenging over time. Development of an instrument to assess the youth-reported burden could aid in preventing T1DM-associated diseases.

**Methods:**

The aim of this study was to translate and validate the Spanish version of the Problem Area in Diabetes Survey–Pediatric version (PAID-Peds). A multicentre, cross-sectional translation and linguistic validation study was performed on a sample of 30 participants aged 8–17 years with a minimum 1-year history of T1DM diagnosed at the Miguel Servet University Hospital in Zaragoza (Aragon, Spain), Ramón y Cajal University Clinical Hospital in Madrid (Spain), and Sant Joan de Déu Hospital in Barcelona (Catalonia, Spain). The qualitative validation consisted of translation into Spanish and back-translation into English of the Paid-Peds survey and subsequent administration to the sample population. Data were gathered on parameters related to sociodemographic characteristics and metabolic control. Validity, feasibility, and test-retest reliability were evaluated. Internal consistency was determined using Cronbach’s alpha coefficient, test-retest reliability by means of interclass correlation, and paired samples using the Wilcoxon W-test. The study was approved by the ethics and research committees at each participating centre.

**Results:**

The study assessed 30 children (46.7% female) with an average age of 13.33 ± 2.98 years; mean age at onset was 5.70 ± 3.62 years, and the mean disease duration was 7.63 ± 4.36 years. The mean score on the PAID-Peds survey was 42.88 ± 17.85. Cronbach’s alpha coefficient was 0.90. Test-retest reliability measured by interclass correlation coefficient was 0.8 (95% CI: 0.63–0.90). No significant differences in total scores were found between test and retest (Wilcoxon W-test: 289; p = 0.051).

**Conclusions:**

The Spanish version of the PAID-Peds survey is a feasible, valid, and reliable instrument to assess the youth-perceived burden of T1DM.

## Introduction

Type 1 diabetes mellitus (T1DM) affects 490,000 children worldwide [1], with 100,000 new cases diagnosed every year [2].

T1DM is asymptomatic in the early stage [3]. Diagnosis is mainly based on blood glucose monitoring and clinical symptoms [4]. Other parameters associated with diabetes are presence of autoantibodies, oral glucose tolerance, and the results of glycated haemoglobin (HbA1c) testing [5].

Following an initial diagnosis of T1DM after debut, the treatment goals include successful initiation of insulin therapy, self-monitoring of blood sugar, and structured, age-appropriate patient education and psychosocial care for the family [6].

Children transitioning to self-management of T1DM may experience difficulties due to a worsening of glycaemic control, especially as they approach adolescence. Suboptimal disease management at this stage is associated with a risk of microvascular complications and a high psychological burden [7, 8].

Diabetes treatment in childhood is complex. Maintaining strict glycaemic control requires lifestyle changes and daily decision-making concerning insulin administration as well as an individual nutrition plan. The challenge posed by these tasks is compounded by everyday worries regarding acute decompensations or future complications of the disease, thereby placing a psychological and psychosocial burden on patients and their caregivers [9].

Young people with DMT1 experience emotional distress related to the daily burden of living with diabetes [10]. Further disease-related problems at this stage of life include possible parental overprotection, issues with body image, eating disorders, and the like. These problematic situations contribute to the psychological vulnerability of young people and may have a negative impact on self-management. Challenges such as these may be overcome by patient strengths including resilience, adaptive processes, or a results-oriented mindset [8].

The Problem Areas in Diabetes (PAID) survey was created and validated in the 1990s to measure and assess diabetes-related burden in adults [11]. In 2015, the survey was adapted for use in young patients aged 8–17 years (PAID-Peds). This instrument may be useful in clinical and research settings as a valid and reliable tool to measure youth-perceived burden of T1DM [12].

According to the scientific evidence in adults, diabetes stress mediates the relationship between depressive symptoms and HbA1c, which suggests the importance of addressing the emotional health of young people with T1DM [13].

In recent years, attention has been paid to the relationship between emotions and health [14]. Low emotional well-being among parents is associated with unsupportive parenting behaviours for diabetes distress and behaviour problems in diabetic youths, which could result in suboptimal HbA1c. This suggests that interventions should be aimed at those families with parents who exhibit emotional distress related to diabetes [15].

## Methods

The aim of this study was to validate the Spanish version of the PAID-Peds survey through a qualitative validation phase and analysis of the psychometric properties of the instrument.

### Design and setting of the study

#### Procedures and stages: qualitative validation

The PAID-Peds survey, originally developed in English by Markowitz et al., is a specific instrument to measure youth-reported burden related to T1DM management [12]. The survey begins with an instructions section followed by 20 items to assess burden over the previous month. There are 5 response options scored on a 0–4 Likert scale (agree to disagree). When applying the questionnaire in the present study, the sample was divided into 2 age groups: 8–12 and 13–18 years.

The study authors contacted Markowitz et al. and the Pediatric, Adolescent and Young Adult Section at the Joslin Diabetes Center (Department of Psychiatry, Harvard Medical School, Boston, MA), who authorised the translation of the instrument into Spanish and subsequent validation.

**Fig. 1:**
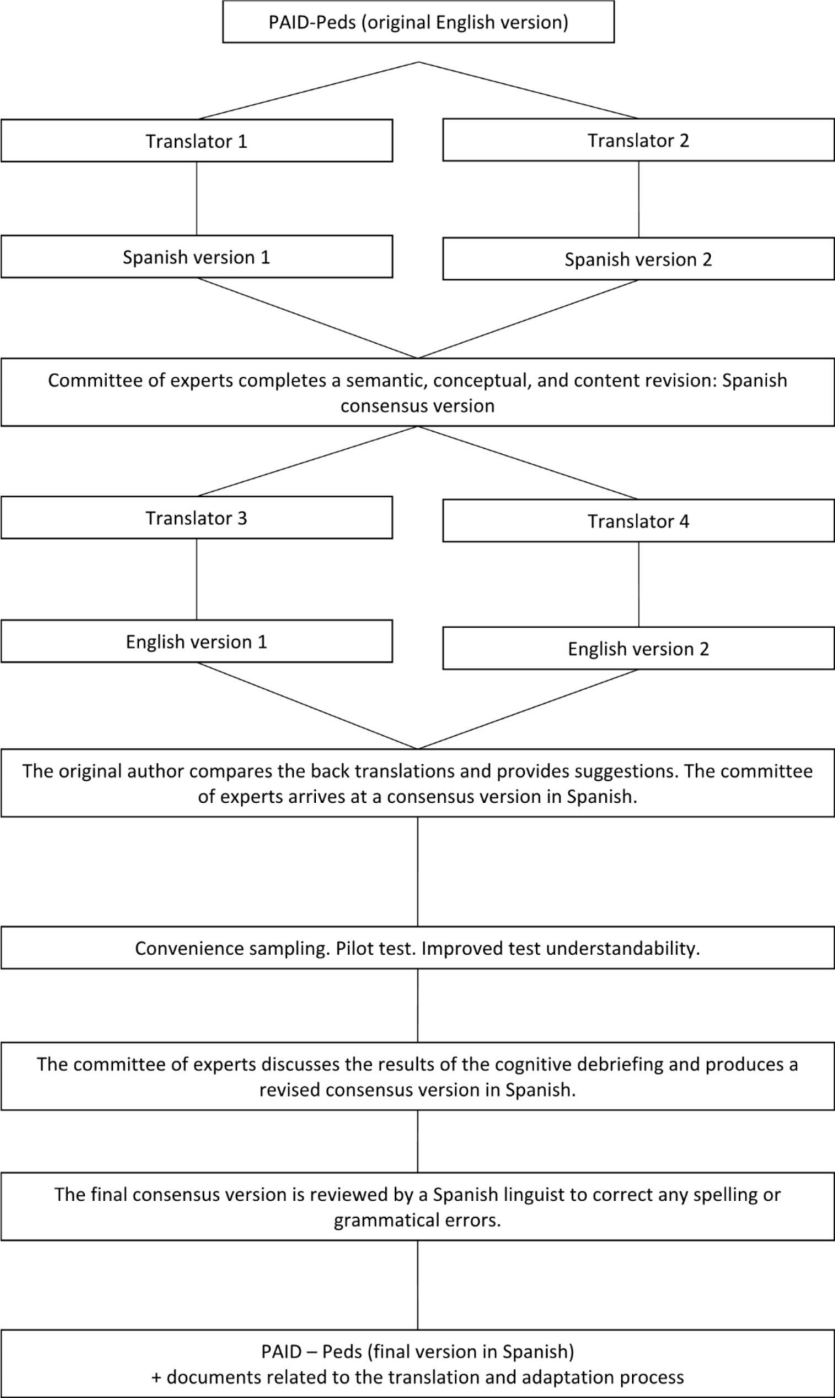
Stages of translation into Spanish, cultural adaptation, and validation of the PAID-Peds® survey

The aim of this stage was to evaluate the face validity and feasibility of the Spanish version. We used an eight-step structured method in accordance with the principles of good practice of the International Society for Pharmacoeconomics and Outcomes Research Task Force for Translation and Cultural Adaptation [16]:

Step 1: Forward translation. Two native Spanish-speaking professional translators independently translated the questionnaire into Spanish; Step 2: Reconciliation and synthesis. Five multidisciplinary specialists in diabetes and endocrinology disorders compared and merged the two translations into one single translation; Step 3: Back-translation. Working independently, 2 native English-speaking professional translators translated the questionnaire into English; Step 4: Comparison and harmonisation of the back translations with the original; Step 5: Cognitive debriefing. Multicentre pilot study of 30 young patients with T1DM selected by consecutive sampling to evaluate the Spanish version of the PAID-Peds survey; Step 6: Review of the cognitive debriefing; Step 7: Proofreading, spelling, and grammar revision; Step 8: Final report (Fig. 1).

### Characteristics of participants

#### Psychometric properties

##### Setting and sampling

An observational, multicentre, cross-sectional validation study.

Inclusion criteria: a sample of 30 subjects aged 8–17 years diagnosed with T1DM for more than one year according to the International Society for Pediatric and Adolescent Diabetes (ISPAD) criteria [5] under treatment with insulin therapy (multiple doses or continuous subcutaneous infusion pump) and under follow-up at the Miguel Servet University Hospital in Zaragoza (Aragón, Spain), the Ramón y Cajal University Clinical Hospital in Madrid (Spain), and the Sant Joan de Déu Hospital in Barcelona (Catalonia, Spain).

Exclusion criteria: Patients diagnosed with disorders associated with intellectual diversity (chromosopathies, autism, cerebral palsy, etc.) and language difficulties.

In addition, the authors of manuscript, as a future line of research, propose a multicentre cross sectional study of 636 population (CI 95% in the same participants’ hospitals).

##### Study variables

Sociodemographic and clinical characteristics were collected for the study. The sociodemographic variables were sex, age, and type of family. The clinical variables were weight; SD weight; height; SD height; body mass index; systolic and diastolic blood pressure; chronic complications; HbA1c (%) at 3, 6, 9, and 12 months prior to the study; glucose management indicator; glucose time in range; year of disease onset; insulin administration type; number of hospitalisations due to ketoacidosis in the previous year; number of episodes of hyperglycaemia treated in the emergency department; history of hypoglycaemia with and without loss of consciousness; hypoglycaemia with loss of consciousness and administration of glucagon; hypoglycaemia without loss of consciousness requiring immediate medical attention; hypoglycaemia without loss of consciousness and with hospitalisation; and other autoimmune disorders.

All patients completed the Spanish version of the PAID-Peds survey. The time required to complete the survey was recorded.

The study was approved by the Ethics and Research Committee of the Autonomous Community of Aragon and the Ramón y Cajal University Hospital of Madrid (C.P.-C.I. P21/425) and the Fundació Sant Joan de Déu (C.I. PIC-33-22). Informed consent for all participants was obtained and patients with age less 16, informed consent was obtained from their respective parent(s)/guardian. All methods were carried out in accordance with relevant guidelines and regulations and Declaration of Helsinki was followed.

### Score and time to complete the questionnaire

Each question was scored on a five-point Likert scale (0: strongly agree; 1: agree; 2: unsure; 3: disagree; 4: strongly disagree). The total score was obtained by reversing the scores of each item and calculating the mean of all non-missing items and multiplying this value by 25 to normalize the total score to a 100-point scale [12]. Patients scoring 41 or higher may be at the level of “emotional burnout” [17].

### Internal consistency

The internal consistency of each question was determined using Cronbach’s alpha index [18], with values of 0.7 or higher considered as acceptable [19].

### Test-retest reliability

The PAID-Peds survey was administered a second time 3 weeks after the first one in the same study population. The test-retest reliability was evaluated using the interclass correlation coefficient and Wilcoxon W-test for paired samples and there were not any losses.

The statistical calculations were performed with Jamovi® statistical software, version 2.3.13. The level of statistical significance was established at a p value of < 0.05.

## Results

### Characteristics of participants

Thirty children with a history of DMT1 of > 1 year participated in the study, 46.7% [14] of whom were male and 53.3% [16] female; 86.7% lived in two-parent families. Mean values for patient characteristics were as follows: age 13.33 ± 2.98 years, age at onset 5.70 ± 3.62 years, history of disease 7.63 ± 4.36 years, weight 48.20 ± 14.86 kg, standard deviation of weight − 0.80 ± 2.84, height 154.12 ± 16.17 centimetres, standard deviation of height − 0.16 ± 1.02, and body mass index 19.71 ± 2.73 (Table 1).

The mean systolic and diastolic blood pressure was 108.54 ± 14.64 and 64.07 ± 6.50 mmHg, respectively, and mean percentage values for HbA1c (current and 3, 6, 9 and 12 previous months) were 7.02 ± 0.90, 6.98 ± 0.75, 6.99 ± 0.75, 7.05 ± 1.10, and 7.13 ± 1.09, respectively. The mean GMI was 7.01 ± 0.76%, current blood glucose test 154.79 ± 31.60 mg/dl, the coefficient of variation (CV) was 39.24 ± 8.22, and TIR (< 50, 50–70, 70–180, 180–250 and > 250) was 1.18 ± 2.55, 3.63 ± 2.65, 65.50 ± 17.06, 20.80 ± 10.38, and 8.90 ± 11.70, respectively.

The average time the participants spent answering the questionnaire was 5.83 ± 3.19 min and the average overall survey score was 42.88 ± 17.85.

**Table 1:** Data on sociodemographic parameters, metabolic control, and total scores and questionnaire completion times for the study population

	Mean	SD	95% confidence interval		Median	IQR
			Lower	Upper		
Age (years)	13.33	2.98	12.22	14.45	13.41	5.64
Age at onset (years)	5.70	3.62	4.35	7.05	4.60	4.42
Duration of T1DM (years)	7.63	4.36	6.00	9.26	6.47	6.53
Weight (Kg)	48.20	14.86	42.65	53.75	50.50	25.48
SD Weight	-0.35	0.96	-0.71	0.01	-0.36	0.96
Height (cm)	154.12	16.17	148.08	160.16	157.65	23.58
SD Height	-0.16	1.02	-0.55	0.22	-0.15	1.11
BMI	19.71	2.73	18.70	20.73	19.90	4.03
SBP (mmHg)	108.54	14.64	102.86	114.21	106.50	12.25
DBP (mmHg)	64.07	6.50	61.55	66.59	62.50	9.50
HbA1c (%)						
Current	7.02	0.90	6.68	7.36	6.70	1.10
Previous 3 months	6.98	0.75	6.69	7.28	6.80	0.90
Previous 6 months	6.99	0.75	6.69	7.29	6.80	1.00
Previous 9 months	7.05	1.10	6.61	7.50	6.75	0.85
Previous 12 months	7.13	1.09	6.71	7.55	6.80	0.73
Current GMI (%)	7.01	0.76	6.73	7.29	6.80	0.90
Current glucose (mg/dl)	154.79	31.60	142.99	166.59	144.00	33.50
CV diabetes (%)	39.24	8.22	36.18	42.31	38.15	10.63
Time in range (%)						
< 54	1.17	2.55	0.22	2.12	0.00	1.00
54–70	3.63	2.65	2.65	4.62	3.00	2.75
70–180	65.50	17.06	59.13	71.87	70.50	20.25
180–250	20.80	10.38	16.92	24.68	18.00	11.50
> 250	8.90	11.70	4.53	13.27	5.00	5.00
Time spent answering the questionnaire	5.83	3.19	4.64	7.02	5.00	2.75
PAID-Peds total score	42.88	17.85	36.21	49.54	41.25	27.19

T1DM: diabetes mellitus type 1; Kg: kilograms; SD: standard deviation; Cm: centimetres; BMI: body mass index; SBP: systolic blood pressure; DBP: diastolic blood pressure; HbA1c: glycated haemoglobin; GMI: glucose management indicator; CV: coefficient of variation; IQR: interquartile range

### Reliability test

The result of the Cronbach’s alpha test was 0.90 (strong) and the rank correlations for each item was (surprise item) 0.29–0.75. The correlation for item 6 was 0.132. The result of Cronbach’s alpha was similar (0.904) after removing the question from the questionnaire (Table 2).

No statistically significant differences were found between the scores obtained in the questionnaire (< 41 normal score; ≥41 emotional distress burnout) and the variables age, age at onset, duration of T1DM, weight, height, BMI, SBP, DBP, HbA1c (current, previous 3, 6, 9 and 12 months), current GMI, current glucose, CV diabetes, time in range (< 50, 50–70, 70–180, 180–250, > 250), and time spent answering the questionnaire.

Strong positive correlations were found between the scores of PAID-Peds and Hb1Ac (Pearson’s test: 0.83; p = 0.04) and coefficient of variation (Pearson’s test: 0.92; p = 0.02).

Regarding the difficulty in understanding the questions, more than 90% of the study participants required no help to complete the questionnaire.

### Test-retest reliability

The participants retook the questionnaire an average of 23.8 ± 9.81 (95% CI: 20.2–27.5) days after the first test and there were not any losses. Mean values for the total retest score and response time were 38.8 ± 18.58 (95% CI: 31.9–45.7) and 4.33 ± 1.94 (95% CI: 3.61–5.06) minutes, respectively. Statistically significant differences between test-retest response time were found (Wilcoxon W-test: 222; p = 0.002). There were no significant differences between total survey scores in test-retest (Wilcoxon W-test: 289; p = 0.051). Test-retest reliability (n = 30) was good, with an interclass correlation of 0.80 (95% CI: 0.63–0.90).

**Table 2:** Test-retests correlation and Cronbach’s alpha test for each question of the PAID-Peds survey Spanish version

Question	Item-rest correlation		Cronbach’s α	
	Test	Retest	Test	Retest
Item 1	0.735	0.828	0.890	0.910
Item 2	0.472	0.638	0.897	0.915
Item 3	0.439	0.433	0.898	0.919
Item 4	0.753	0.788	0.889	0.910
Item 5	0.382	0.610	0.900	0.915
Item 6	0.132	0.496	0.904	0.918
Item 7	0.733	0.808	0.890	0.911
Item 8	0.684	0.624	0.891	0.915
Item 9	0.712	0.739	0.890	0.912
Item 10	0.743	0.768	0.890	0.912
Item 11	0.420	0.526	0.898	0.917
Item 12	0.492	0.540	0.897	0.917
Item 13	0.627	0.470	0.893	0.918
Item 14	0.598	0.616	0.894	0.915
Item 15	0.446	0.486	0.898	0.918
Item 16	0.294	0.351	0.901	0.922
Item 17	0.628	0.855	0.893	0.910
Item 18	0.334	0.480	0.901	0.918
Item 19	0.300	0.265	0.901	0.923
Item 20	0.476	0.364	0.897	0.921

## Discussion

The main objective of this study was to translate, culturally adapt, and validate the PAID-Peds survey by means of a staged qualitative validation and analysis of the psychometric properties of the instrument.

Several questionnaires assess the burden and health-related quality of life of diabetes in adults, mainly type 2 diabetes mellitus [11, 20, 21]. The PAID survey, created in 1990, is the most widely used international scale to assess the stress associated with diabetes in adults [15]; diabetes has been associated with dysfunctional coping styles, poorer quality of life, and depressive symptoms [22]. However, there is currently no instrument in Spanish to assess the emotional burden of the disease among diabetic children. The PAID survey was therefore adapted for the paediatric age group (PAID-Peds) [23, 24] and has clinical and research utility as a valid and acceptable measure of the type of burden perceived by young people aged 8–17 years with T1DM [25].

The original English questionnaire yielded a Cronbach’s alpha of 0.94 [12]. In the process of translation into Spanish and cultural adaptation and validation, a similar internal consistency was obtained [19] and the intraclass correlation coefficient for the test-retest was higher than that of the English counterpart.

Psychological problems are usually underdiagnosed in people with diabetes [26]; therefore, few studies examine emotional well-being and diabetes-associated stress in children.

Diabetes distress refers to the negative emotional impact of living with diabetes. Its clinical importance is tangible, as it is associated with poorer adherence, worse self-care, and suboptimal glycaemic control [9, 10, 27]. Higher glycosylated haemoglobin (HbA1c) values and reduced self-management behaviours are often associated with lower quality of life, higher prevalence of depressive symptoms, and significant diabetes distress [8]. Assessment of the psychological, social, and emotional impact of diabetes in children and adolescents should be routinely included to detect patient needs and barriers to effective self-management, as recommended by ISPAD [28], and there is evidence of a relationship between a higher perceived quality of life and lower HB1Ac [29].

Therapeutic diabetes education (TDE), which is recommended by the World Health Organisation and the Diabetes Education Study Group, is a necessary educational process that is integrated in treatment approaches for diabetes. TDE aims to provide diabetics and their families with the competencies (i.e., knowledge, skills and attitudes) and support necessary to self-manage their disease. When successful, TDE helps patients understand their illness and the bases of treatment, to integrate treatment into their daily lives, as well as to prevent, recognise, and act in acute risk situations and prevent cardiovascular risk factors [30].

Detection of diabetes-specific emotional burden in childhood favours early interventions to reduce distress and prevent the worsening of distress, burnout, depressive symptoms, and subsequent poor diabetes self-management [23].

Structured diabetes education comprises distinct and complex goals aimed at empowering patients to manage diabetes and to overcome the emotional challenges associated with their chronic disease. However, the many components of diabetes education cannot currently be assessed separately [31].

## Conclusions

The psychometric properties of the Spanish version of the Problem Areas in Diabetes Survey–Pediatric Version (PAID-Peds) demonstrate that the tool has adequate feasibility, validity, and reliability for use in both clinical practice and research in Spanish-speaking people.

## Data Availability

The datasets generated and/or analysed during the current study are available from the corresponding author on reasonable request.
